# Relationship Between Age-Related Changes in Skeletal Muscle Mass and Physical Function: A Cross-Sectional Study of an Elderly Japanese Population

**DOI:** 10.7759/cureus.24260

**Published:** 2022-04-18

**Authors:** Hiroaki Iwase, Shin Murata, Hideki Nakano, Kayoko Shiraiwa, Teppei Abiko, Akio Goda, Koji Nonaka, Kunihiko Anami, Jun Horie

**Affiliations:** 1 Department of Physical Therapy, Faculty of Rehabilitation Science, Kobe International University, Kobe, JPN; 2 Department of Physical Therapy, Faculty of Health Sciences, Kyoto Tachibana University, Kyoto, JPN; 3 Department of Rehabilitation, Faculty of Health Sciences, Naragakuen University, Nara, JPN

**Keywords:** japanese, physical function, grip strength, skeletal muscle mass, elderly people

## Abstract

Skeletal muscle mass and muscle strength are positively correlated, but the relationship between grip strength and global muscle strength is controversial. This study aimed to clarify the changes in site-specific skeletal muscle mass by age group and determine the relationship between site-specific, age-related changes in skeletal muscle mass and physical function in community-dwelling elderly people in Japan. The participants were divided into age groups of five-year intervals (65-69 years, 70-74 years, 75-79 years, and ≥80 years) and were also categorized by sex. The skeletal muscle mass of the upper limbs, lower limbs, and trunk was measured using multifrequency bioelectrical impedance analyzers (InBody 430 (Biospace Co., Ltd., Seoul, Korea) and InBody 470 (InBody Japan Inc., Tokyo, Japan)). For physical function assessment, we measured grip strength, quadriceps strength, sit-up count, sit-and-reach distance, and standing time on one leg with eyes open and performed the timed up and go (TUG) test. The results showed that skeletal muscle mass decreased with age regardless of sex at all measured sites. Furthermore, a partial correlation analysis adjusted for age, physical constitution, and the presence/absence of exercise habits revealed that the highest correlation was between skeletal muscle mass in all sites and grip strength. Thus, monitoring grip strength may be used as a representative of systemic skeletal mass even in Japanese people.

## Introduction

Changes in body composition are strongly associated with physical disability among the elderly [1,2]. Previous studies reported that skeletal muscle mass decreases at a rate of approximately 5% every 10 years after the age of 30 years and that the rate of loss accelerates after the age of 60 years [3,4]. Furthermore, elderly people aged ≥75 years reportedly lose skeletal muscle mass at an annual rate of approximately 1% [5]. According to previous imaging studies on the loss of skeletal muscle mass, greater and more rapid age-associated loss of skeletal muscle mass occurs in the lower limbs than in the upper limbs [6-8].

Skeletal muscle mass and muscle strength are positively correlated [9]. Many previous studies in elderly populations utilized grip strength as a measure of global muscle strength. Porto et al. observed a significant association between grip strength and muscle strength pertaining to 10 muscle groups [10]. However, it has been reported that skeletal muscle mass alone does not determine muscle strength [11]. Moreover, the authors of another study suggested caution when referring to the representativeness of grip strength as a predictor of global muscle strength [12]. Thus, no consensus has been reached on this matter.

As skeletal muscle mass varies according to race, it is necessary to investigate age-associated changes in skeletal muscle mass and the relationship between skeletal muscle mass and physical function including muscle strength in a Japanese population. Therefore, this study aimed to elucidate age-associated changes in site-specific skeletal muscle mass and determine the relationship between site-specific skeletal muscle mass and physical function in community-dwelling elderly Japanese people.

## Materials and methods

Participants

This was a cross-sectional study. We recruited participants from among the elderly people living in Yasu City, Shiga Prefecture, who were registered with a local program intended to prevent long-term care dependency and provide emotional support. Among the elderly people aged ≥65 years who took part in the annual physical fitness tests between 2015 and 2019, we recruited those with no long-term care requirement certifications, history of central nervous system (CNS) disease, or suspected cognitive impairment (Mini-Mental State Examination ≧ 24). Those who participated for multiple years adopted the data of the year they participated for the first time. The participants were divided into age groups of five-year intervals (65-69 years, 70-74 years, 75-79 years, and ≥80 years) and categorized by sex. Participant characteristics were recorded.

Physical fitness tests were conducted under the supervision of public health nurses. The participants received a full explanation in advance of the measurements to be acquired and how the data would be managed. All participants provided written informed consent. This study was approved by the Research Ethics Committee of Kyoto Tachibana University (approval number: 17 - 14).

Assessments

In addition to skeletal muscle mass, we evaluated grip strength, quadriceps strength, sit-up count, sit-and-reach distance, and standing time on one leg with eyes open. The timed up and go (TUG) test was also performed.

For the measurement of skeletal muscle mass, we used multifrequency bioelectrical impedance analyzers (InBody 430 (Biospace Co., Ltd., Seoul, Korea) and InBody 470 (InBody Japan Inc., Tokyo, Japan)). The muscle mass (kg) of the upper and lower limbs, which constitute a proportion of the lean soft tissue mass (kg), was calculated as the total of both (the right and left) upper and lower limbs, respectively, excluding the muscle mass of the trunk.

Grip strength was measured using a digital grip dynamometer (T.K.K.58401, Takei Scientific Instruments Co., Ltd., Niigata, Japan) [13]. The grip width was adjusted such that the proximal interphalangeal joint of the index finger was flexed at 90°. The participants were instructed to stand in an upright position with the feet placed shoulder width apart and arms hanging by the sides of the body. They were then instructed to grip with maximum effort without the dynamometer touching their bodies. Two measurements were taken for the right-hand and left-hand grips, and the maximum values (kg) were considered to be representative.

A handheld dynamometer (μTas F-1, Anima Corp., Tokyo, Japan) was used to measure quadriceps strength. The measurements were taken while the participants were in a sitting position with knees flexed at 90° [14]. Two measurements were acquired for each lower limb. The maximum values (kg) were considered to be representative.

Sit-ups were performed in the supine position with both arms crossed in front of the chest and both knees flexed at 90°. We counted the number of times that both elbows touched both thighs during a period of 30 seconds.

To measure the sit-and-reach distance, we used a specialized digital device (T.K.K.5412, Takei Scientific Instruments Co., Ltd., Niigata, Japan). Two measurements were acquired, and the maximum values (cm) were considered to be representative.

The standing time on one leg with eyes open was measured using a digital stopwatch with an upper limit of 120 seconds. Two measurements were acquired for each leg, and the longest time was regarded as a representative value. The participants were instructed to barefoot and keep both upper limbs lightly touching the sides of the body and maintain their line of sight at a fixation point ahead, 2 m above the ground.

The TUG test was conducted based on the Shumway-Cook method [15]. At the beginning of the measurement, the participants sat on a chair with their backs leaning into the backrest and their hands on their knees. At the signal of the examiner, the participants were instructed to stand up, walk 3 m forward as quickly as possible, cross a line marked on the floor, turn around, walk back, and sit back down. The time required for the participants to perform these actions was measured with a digital stopwatch. The test was performed twice, and the shortest time (seconds) was regarded as a representative value.

Statistical analysis

To evaluate skeletal muscle mass according to age, we first examined the interaction between skeletal muscle mass at different sites (upper limbs, lower limbs, and trunk) and sex. As a result, an interaction was established for all items; hence, a trend test by sex was performed for the comparison of skeletal muscle mass according to age. To clarify the relationship between site-specific skeletal muscle mass and physical function, we performed a partial correlation analysis by gender using age, body mass index (BMI), and the presence/absence of exercise habits (at least twice a week with each session lasting approximately 30 minutes), which have been reported to be related to skeletal muscle mass in previous studies, as covariates. SPSS version 25 (IBM Corp., Armonk, NY, USA) was used for all analyses. The significance level was set to 5% in all analyses. Statistical significance was determined by two-tailed tests.

## Results

Participant characteristics

Among the elderly persons who participated in the physical fitness tests, 684 people (148 men and 536 women) were included in the analysis after the exclusion of those who met the exclusion criteria.

Table 1 presents participant attributes by sex. The mean age was higher among men than among women. However, there was no difference in physical constitution (BMI) between men and women. There was also no sex difference among participants receiving treatment for hypertension. However, a higher proportion of women were receiving dyslipidemia treatment, and a higher proportion of men were receiving diabetes treatment.

**Table 1:** Participant attributes Data shown as mean (standard deviation) and % BMI: body mass index

	Total		Men		Women		p-value
	n=684		n=148		n=536		
Age (years)	73.2	(5.6)	74.6	(5.1)	72.8	(5.6)	<0.001
Height (cm)	154.2	(7.9)	164.9	(5.5)	151.2	(5.5)	<0.001
Weight (kg)	53.4	(8.6)	62	(7.2)	51	(7.3)	<0.001
BMI (kg/m^2^)	22.4	(2.9)	22.8	(2.3)	22.3	(3)	0.044
Hypertension treatment (%)	35.8		35.8		35.8		0.998
Dyslipidemia treatment (%)	16.1		8.1		18.3		0.003
Diabetes treatment (%)	8.2		15.5		6.2		<0.001
Educational history (years)	11.9	(2.3)	12.8	(2.8)	11.6	(2)	<0.001

Site-specific skeletal muscle mass and physical function by age group

Table 2 shows the results of the trend tests by sex for the measurements of site-specific skeletal muscle mass and physical function. The trend test revealed that the skeletal muscle mass in the upper limbs, lower limbs, and trunk decreased with age in both men and women. In terms of physical function, grip strength, quadriceps strength, and standing time on one leg with eyes open decreased with age in both men and women. The time required to complete the TUG test increased with age among both men and women. The sit-up count decreased with age among men but not among women. No age-associated change was observed in the sit-and-reach distance among men or women.

**Table 2:** Site-specific skeletal muscle mass and physical function by age group Data shown as mean (standard deviation) TUG: timed up and go test

	Men									Women								
	65-69 years		70-74 years		75-79 years		80 years or older		Trend test	65-69 years		70-74 years		75-79 years		80 years or older		Trend test
	n=24		n=46		n=52		n=22		p-value	n=142		n=179		n=115		n=69		p-value
Body composition																		
Skeletal muscle mass of the upper limbs (kg)	5.3	(0.8)	5.3	(0.9)	5.1	(0.7)	4.8	(0.7)	0.012	3.4	(0.5)	3.2	(0.6)	3.2	(0.5)	3	(0.5)	<0.001
Skeletal muscle mass of the lower limbs (kg)	15.9	(1.9)	15.2	(2.1)	15.1	(2)	14.8	(2.1)	0.024	11	(1.3)	10.3	(1.5)	10.1	(1.3)	9.8	(1.4)	<0.001
Skeletal muscle mass of the trunk (kg)	22	(2.3)	21.8	(2.6)	21.1	(1.9)	20.2	(2.1)	0.003	16	(1.7)	15.2	(1.8)	15.1	(1.6)	14.3	(1.6)	<0.001
Physical function																		
Grip strength (kg)	39.4	(6.2)	36.5	(5.3)	35.3	(5.1)	33.5	(5.8)	<0.001	24.8	(3.6)	23.7	(3.7)	23.2	(4.1)	23.6	(3.8)	<0.001
Quadriceps strength (kg)	30.1	(7.2)	26.7	(5.8)	26.4	(6.1)	26	(8.5)	0.088	21.6	(5)	20.3	(4.3)	18.8	(4.4)	16.8	(4.3)	<0.001
Sit-up count (times)	17.3	(4.7)	12.1	(7.7)	9.9	(6.5)	8.9	(6.4)	<0.001	8.3	(6.7	6.8	(5.9)	8	(6.8)	6.1	(6.1)	0.167
Sit-and-reach distance (cm)	32.8	(8.7)	30.8	(10.3)	29.9	(9.1)	31.3	(9.8)	0.371	36.4	(8)	35.9	(8.5)	35.9	(7.9)	35.2	(8.9)	0.411
Standing time on one leg with eyes open (seconds)	51.3	(39.7)	47.7	(38)	29.6	(27.1)	23.5	(26.2)	<0.001	59.1	(38.9)	51.1	(38.2)	34.3	(28.4)	20.8	(23.7)	<0.001
TUG (seconds)	5.1	(0.9)	5.7	(1.1)	5.9	(1)	6.5	(1.3)	<0.001	5.5	(0.8)	5.7	(0.7)	6.2	(1.1)	7.1	(1.9)	<0.001

Relationship between skeletal muscle mass and physical function

Table 3 indicates the results of the partial correlation analysis by sex, in which age, BMI, and the presence/absence of exercise habits were regarded as covariates. In men, only grip strength was significantly associated with skeletal muscle mass in the upper limbs, lower limbs, and trunk (upper limbs: r=0.43, p<0.01; lower limbs: r=0.36, p<0.01; trunk: r=0.45, p<0.01). In women, all physical function parameters were significantly associated with skeletal muscle mass in the upper limbs, lower limbs, and trunk. However, grip strength had the strongest association with skeletal muscle mass in each of the body parts (upper limbs: r=0.64, p<0.01; lower limbs: r=0.54, p<0.01; trunk: r=0.64, p<0.01).

**Table 3:** Relationship between site-specific skeletal muscle mass and physical function Partial correlation analysis: adjusted for age, BMI, and exercise habits (yes/no) TUG: timed up and go test **: p<0.01, *: p<0.05

	Men				Women		
	n=148				n=536		
	Upper limbs	Lower limbs	Trunk	Upper limbs		Lower limbs	Trunk
Grip strength	0.43**	0.36**	0.45**	0.64**		0.54**	0.64**
Quadriceps strength	0.05	0.12	0.06	0.21**		0.17**	0.20**
Sit-up count	0.08	0.09	0.06	0.29**		0.19**	0.24**
Sit-and-reach distance	0.07	0.07	0.06	0.26**		0.23**	0.25**
Standing time on one leg with eyes open	0.01	-0.01	0.02	0.11*		0.13*	0.11*
TUG	-0.08	-0.07	-0.09	-0.11*		-0.16**	-0.14*

## Discussion

We studied age-associated changes in site-specific skeletal muscle mass in Japanese elderly people and examined the relationship between site-specific skeletal muscle mass and physical function. We found that the skeletal muscle mass of the upper limbs, lower limbs, and trunk tended to decrease with age in both men and women. Furthermore, the physical function parameter that had the strongest association with age-related loss of skeletal muscle mass in both men and women was grip strength.

It has been reported that skeletal muscle mass decreases with age and that skeletal muscle mass is higher among men than among women at any age [8,16]. Our finding that the skeletal muscle mass of the upper limbs, lower limbs, and trunk decreases with age is thus consistent with the literature. Although the mechanism of age-related loss of skeletal muscle mass has not been elucidated, it may be related to an age-related decrease in the number of motor nerves, changes in the neuromuscular junction, and a decrease in growth and sex hormone production [6,16,17].

We also examined the relationship between site-specific skeletal muscle mass (upper limbs, lower limbs, and trunk) and physical function. As skeletal muscle mass generally depends on physical constitution (e.g., height, weight, and BMI), when conducting a cross-sectional study of age-related changes, the effect of physical constitution cannot be ignored. In this study, we conducted a partial correlation analysis of the relationship between skeletal muscle mass and physical function. In the analysis, age, BMI, and the presence/absence of exercise habits were regarded as covariates. The analysis revealed a positive correlation between grip strength and skeletal muscle mass at each of the sites measured in men. Furthermore, in women, correlations were established between the skeletal muscle mass at all of the sites measured and all of the physical function parameters. Notably, grip strength had the strongest correlation with skeletal muscle mass at all sites among women.

As grip strength is easy to measure, it is a commonly used evaluation method [18] and is regarded as a measure of global muscle strength. It is associated with elbow flexor strength (r=0.64), knee extensor strength (r=0.53), trunk extensor strength (r=0.52), and trunk flexor strength (r=0.44) [19]. Moreover, grip strength is closely linked to both lower-limb muscle strength and the cross-sectional area of the lower-limb muscles and is also associated with walking ability [20]. Further, reduced grip strength is associated not only with a decline in the ability to perform activities of daily living but also with a decline in cognitive function [21]. Reduced grip strength is also linked to chronic diseases (e.g., diabetes and hypertension) [22], ischemic heart disease [23], depression [24], and increased mortality risk [25,26]. Thus, grip strength measurement is highly valuable in the assessment of physical function in elderly people [10,26]. From these facts, it was suggested that grip strength may be used as a representative of whole-body skeletal muscle mass even in Japanese.

A limitation of this study is that the participants comprised elderly people who were sufficiently independent to take part in the health support program organized by their local city. That is, the participants of this study were health-conscious elderly people. To generalize the findings of this study, it may be necessary to survey and analyze elderly people who do not or cannot take part in a health support program. Furthermore, this was a cross-sectional study. Changes that occur in individuals’ skeletal muscle mass and physical function as they age cannot be tracked via a cross-sectional study. In addition, skeletal muscle mass and neurological factors are included in muscle strength, but neurological factors have not been investigated in this study. Therefore, a future longitudinal study is warranted.

## Conclusions

We investigated the relationship between skeletal muscle mass and physical function in 684 Japanese general elderly people. As a result, skeletal muscle mass decreased with age. In addition, as the age increased, muscle strength, balance ability, and walking ability decreased. Among various physical functions, grip strength had the highest correlation coefficient with skeletal muscle mass. Our findings suggest that grip strength may be used as a representative of systemic skeletal mass even in Japanese people.

## References

[1] Visser M, Harris TB, Langlois J (1998). Body fat and skeletal muscle mass in relation to physical disability in very old men and women of the Framingham Heart Study. J Gerontol A Biol Sci Med Sci.

[2] Visser M, Langlois J, Guralnik JM (1998). High body fatness, but not low fat-free mass, predicts disability in older men and women: the Cardiovascular Health Study. Am J Clin Nutr.

[3] Lexell J, Taylor CC, Sjöström M (1988). What is the cause of the ageing atrophy? Total number, size and proportion of different fiber types studied in whole vastus lateralis muscle from 15- to 83-year-old men. J Neurol Sci.

[4] Lexell J (1995). Human aging, muscle mass, and fiber type composition. J Gerontol A Biol Sci Med Sci.

[5] Mitchell WK, Williams J, Atherton P, Larvin M, Lund J, Narici M (2012). Sarcopenia, dynapenia, and the impact of advancing age on human skeletal muscle size and strength; a quantitative review. Front Physiol.

[6] Janssen I, Heymsfield SB, Wang ZM, Ross R (2000). Skeletal muscle mass and distribution in 468 men and women aged 18-88 yr. J Appl Physiol (1985).

[7] Borkan GA, Hults DE, Gerzof SG, Robbins AH, Silbert CK (1983). Age changes in body composition revealed by computed tomography. J Gerontol.

[8] Gallagher D, Visser M, De Meersman RE (1997). Appendicular skeletal muscle mass: effects of age, gender, and ethnicity. J Appl Physiol (1985).

[9] Reed RL, Pearlmutter L, Yochum K, Meredith KE, Mooradian AD (1991). The relationship between muscle mass and muscle strength in the elderly. J Am Geriatr Soc.

[10] Porto JM, Nakaishi AP, Cangussu-Oliveira LM, Freire Júnior RC, Spilla SB, Abreu DC (2019). Relationship between grip strength and global muscle strength in community-dwelling older people. Arch Gerontol Geriatr.

[11] Beliaeff S, Bouchard DR, Hautier C, Brochu M, Dionne IJ (2008). Association between muscle mass and isometric muscle strength in well-functioning older men and women. J Aging Phys Act.

[12] Felicio DC, Pereira DS, Assumpção AM (2014). Poor correlation between handgrip strength and isokinetic performance of knee flexor and extensor muscles in community-dwelling elderly women. Geriatr Gerontol Int.

[13] Abe T, Yaginuma Y, Fujita E, Thiebaud RS, Kawanishi M, Akamine T (2016). Associations of sit-up ability with sarcopenia classification measures in Japanese older women. Interv Med Appl Sci.

[14] Bohannon RW (1986). Test-retest reliability of hand-held dynamometry during a single session of strength assessment. Phys Ther.

[15] Shumway-Cook A, Brauer S, Woollacott M (2000). Predicting the probability for falls in community-dwelling older adults using the timed up & go test. Phys Ther.

[16] Baumgartner RN, Waters DL, Gallagher D, Morley JE, Garry PJ (1999). Predictors of skeletal muscle mass in elderly men and women. Mech Ageing Dev.

[17] Ho KY, Evans WS, Blizzard RM (1987). Effects of sex and age on the 24-hour profile of growth hormone secretion in man: importance of endogenous estradiol concentrations. J Clin Endocrinol Metab.

[18] FI MB, BI JE (1946). Standardization of a test of hand strength. J Appl Psychol.

[19] Rantanen T, Era P, Kauppinen M, Heikkinen E (1994). Maximal isometric muscle strength and socioeconomic status, health, and physical activity in 75-year-old persons. J Aging Phys Activity.

[20] Lauretani F, Russo CR, Bandinelli S (2003). Age-associated changes in skeletal muscles and their effect on mobility: an operational diagnosis of sarcopenia. J Appl Physiol (1985).

[21] Arokiasamy P, Selvamani Y (2018). Age, socioeconomic patterns and regional variations in grip strength among older adults (50+) in India: Evidence from WHO's Study on Global Ageing and Adult Health (SAGE). Arch Gerontol Geriatr.

[22] Stenholm S, Tiainen K, Rantanen T, Sainio P, Heliövaara M, Impivaara O, Koskinen S (2012). Long-term determinants of muscle strength decline: prospective evidence from the 22-year mini-Finland follow-up survey. J Am Geriatr Soc.

[23] Juul A, Scheike T, Davidsen M, Gyllenborg J, Jørgensen T (2002). Low serum insulin-like growth factor I is associated with increased risk of ischemic heart disease: a population-based case-control study. Circulation.

[24] Zasadzka E, Pieczyńska A, Trzmiel T, Kleka P, Pawlaczyk M (2021). Correlation between handgrip strength and depression in older adults-a systematic review and a meta-analysis. Int J Environ Res Public Health.

[25] Roubenoff R, Parise H, Payette HA (2003). Cytokines, insulin-like growth factor 1, sarcopenia, and mortality in very old community-dwelling men and women: the Framingham Heart Study. Am J Med.

[26] Bohannon RW (2019). Grip strength: an indispensable biomarker for older adults. Clin Interv Aging.

